# Thioredoxin Reductase Inhibitors as Potential Antitumors: Mercury Compounds Efficacy in Glioma Cells

**DOI:** 10.3389/fmolb.2022.889971

**Published:** 2022-06-23

**Authors:** Vanessa Pires, Isabella Bramatti, Michael Aschner, Vasco Branco, Cristina Carvalho

**Affiliations:** ^1^ Research Institute for Medicines (iMed.ULisboa), Faculty of Pharmacy, Universidade de Lisboa, Lisboa, Portugal; ^2^ Department of Molecular Pharmacology, Albert Einstein College of Medicine, Bronx, NY, United States; ^3^ Centro de Investigação Interdisciplinar Egas Moniz (CiiEM), Instituto Universitário Egas Moniz (IUEM), Caparica, Portugal

**Keywords:** glioblastoma, thioredoxin reductase, thioredoxin, thimerosal (thiomersal), ethylmercury

## Abstract

Glioblastoma multiforme (GBM) is the most aggressive and common form of glioma. GBM, like many other tumors, expresses high levels of redox proteins, such as thioredoxin (Trx) and thioredoxin reductase (TrxR), allowing tumor cells to cope with high levels of reactive oxygen species (ROS) and resist chemotherapy and radiotherapy. Thus, tackling the activity of these enzymes is a strategy to reduce cell viability and proliferation and most importantly achieve tumor cell death. Mercury (Hg) compounds are among the most effective inhibitors of TrxR and Trx due to their high affinity for binding thiols and selenols. Moreover, organomercurials such as thimerosal, have a history of clinical use in humans. Thimerosal effectively crosses the blood–brain barrier (BBB), thus reaching effective concentrations for the treatment of GBM. Therefore, this study evaluated the effects of thimerosal (TmHg) and its metabolite ethylmercury (EtHg) over the mouse glioma cell line (GL261), namely, the inhibition of the thioredoxin system and the occurrence of oxidative cellular stress. The results showed that both TmHg and EtHg increased oxidative events and triggered cell death primarily by apoptosis, leading to a significant reduction in GL261 cell viability. Moreover, the cytotoxicity of TmHg and ETHg in GL261 was significantly higher when compared to temozolomide (TMZ). These results indicate that EtHg and TmHg have the potential to be used in GBM therapy since they strongly reduce the redox capability of tumor cells at exceedingly low exposure levels.

## Introduction

Glioblastoma multiforme (GBM) is the most aggressive and common form of glioma, being responsible for 3–4% of all cancer-related deaths (Carlsson et al., 2014; Kaya et al., 2016). The risk of prevalence increases with age, being more common in people aged 60–79 years (Kyani et al., 2018). Upon diagnosis, median survival is 12–18 months with only 3–5% of patients surviving more than 3 years (Tykocki and Eltayeb, 2018). Temozolomide (TMZ) is frequently used in GBM treatment in combination with surgery and radiation, but the increase in the average life expectancy is very limited (2.5 months) (Minniti et al., 2008; Brunetti et al., 2019). Therefore, new therapeutic options for GBM patients, particularly repurposing of already licensed drugs, are being considered a major pathway to finding more effective therapies (Carlsson et al., 2014).

A major factor contributing to therapy resistance is the overexpression of redox-active systems, namely, the thioredoxin system which comprises thioredoxin (Trx), the selenoenzyme thioredoxin reductase (TrxR), and NADPH (Arnér and Holmgren, 2006).

The overexpression of the thioredoxin system allows tumors to cope with increased production of ROS and maintain functions related to cancer hallmarks (Trachootham et al., 2009; Branco et al., 2020). Indeed, in addition to an antioxidant function, the thioredoxin system regulates protein repair, DNA synthesis, cell signaling, and ASK-1 mediated apoptosis (Arnér and Holmgren, 2006; Hashemy, 2011).

Since the thioredoxin system is overexpressed in tumor cells, its inhibition appears as a promising therapeutic strategy to increase ROS levels and oxidative stress and induce GBM cell apoptosis (Nordberg et al., 2001; Watson et al., 2004; Branco et al., 2020).

Among the known inhibitors of the thioredoxin system, mercury (Hg) compounds are the most effective due to their high affinity for binding to thiols and selenols (Branco et al., 2017). TrxR is highly sensitive to Hg inhibition due to the reactivity and position of the Sec residue in the open C-terminal of the TrxR active site. Trx is also a target of mercury compounds, which bind Cys32 and Cys35 at the active site as well as structural Cys at positions 62, 69, and 73 (Carvalho et al., 2008).

Thimerosal (TmHg) has a history of clinical use, namely as a preservative in vaccines. Once in cells thimerosal releases ethylmercury (EtHg), which is highly effective in inhibiting TrxR at sub-cytotoxic concentrations (Rodrigues et al., 2015). Moreover, these compounds effectively cross the blood–brain barrier (BBB) and accumulate in the brain (Burbacher et al., 2005; Blanuša et al., 2012; Lohren et al., 2016; Afsordeh et al., 2019; Kern et al., 2020), which is fundamental in the context of GBM therapy. The Joint FAO (Food and Agriculture Organization of the United Nations)/WHO Expert Committee on Food Additives (JECFA) 2004 established that the safe concentration of methylmercury intake, without the appearance of neurological disorders, is 1.6 µg/Kg b.w./week of body weight^1^. Data in *Macaca fascicularis* report that EtHg accumulation in the brain is lower than during exposure to MeHg (Burbacher et al., 2005). Our aim is to verify if thimerosal (pro-drug) and/or its metabolite EtHg are drug candidates for further investigation in clinical studies directed at glioblastoma treatment, especially progressive or recurrent high-grade glioma. Therefore, this study evaluated the effect of TmHg and EtHg on the viability of GL261 cells, assessing the effects on the thioredoxin system, mitochondrial redox function, ROS production, and apoptosis. Most importantly, we compared the effects of mercurial compounds, namely their efficacy in reducing tumor cell viability, to the efficacy of standard contemporary clinical treatment with TMZ.

## Materials and Methods

### Cell Culture

Mouse glioma cells (GL261) were kindly provided by Professor Maria da Conceição Pedroso de Lima (Center for Neuroscience and Cell Biology, University of Coimbra, Portugal). This cell line was chosen since it is a very common model for GBM studies, presenting normal features of an astrocytoma (Strong et al., 2018) and is resistant to TMZ therapy similarly to human GBM (Haddad et al., 2021). Moreover, xenografts of these cells display histopathological features that closely resemble the human GBM (Yi et al., 2013). GL261 cells were cultured in DMEM (Dulbecco’s Modified Eagle’s Medium; Gibco) containing 4.5 g/L of glucose, Glutamax, 25 mM HEPES and supplemented with 10% of heat-inactivated (30 min, 56°C) fetal bovine serum (FBS; Biochrome) and 1% penicillin/streptomycin (Gibco) in a humidified incubator at 37°C and 5% CO_2_.

### Cell Viability

#### MTT Assay

The effect of TmHg and EtHg on the viability of GL261 and N9 cells was evaluated by the MTT assay as previously described (Branco et al., 2014). Briefly, cells (5 × 10^3^) were seeded in 96-well plates (Nunc^®^) and incubated at 37°C for 24 h to allow proper adherence to plates. Afterwards, TmHg and EtHg (0, 0.5, 1, 2.5, and 5 μM) were added to wells. The same procedure was carried out to evaluate the effect of TMZ on the cellular viability of GL261 and DMSO at a final concentration of 0.1% was added as a control group. For co-exposure, the addition of the mercury compounds (EtHg—1 and 2 μM; TmHg—1 and 2 µM) under study followed the addition of TMZ (200 µM).

Cell viability was determined at the time of addition of the compounds (0 h) and after 24, 48, and 72 h of exposure by adding MTT to a final concentration of 400 μg/ml per well and incubating at 37°C for 4 h. After incubation, the medium was removed, and the formazan crystals were dissolved in a 4:1 DMSO/glycine buffer (pH 10.5). After shaking for 15 min, the viability was evaluated through the measurement of formazan absorption at 550 nm on a microplate reader (Zenyth 3100, Anthos Labtec Instruments) (Branco et al., 2014).

The IC_50_ is the concentration of a compound that decreases MTT reduction by 50% relative to the non-treated control at each time point.

#### LDH Assay

Cells were seeded in 96-well plates for 24 h prior to the addition of TmHg, EtHg (1 µM), TMZ (200, 400, and 800 µM), and for the 48 h end point the co-exposure of TMZ 200 µM with 1 µM EtHg and 1 µM TmHg. After exposure for 24 and 48 h, the supernatant was transferred to a fresh plate and a lysis buffer (10×) (Thermo Scientific™ Pierce™) was added to wells containing cells and incubated at 37°C for 45 min. LDH activity was evaluated using LDH Cytotoxicity Assay Kit from Thermo Scientific™ Pierce™ in the supernatant and lysate by the addition of lactate (substrate), a tetrazolium salt, and NAD^+^ (cofactor) (Reaction Mixture), according to the manufacturer’s instructions. Following 30 min of incubation in the dark at room temperature, LDH release was assessed by measuring the absorbance at 490 and 680 nm in a microplate reader (Omega BMG LABTECH). The release of LDH was quantified as the ratio between the amount in the supernatant and total LDH (supernatant + lysate) (Branco et al., 2017).

### Preparation of Cell Lysates

Total cell lysates were prepared according to previous protocols (Branco et al., 2014). 1 × 10^6^ cells were plated in 10 mm^2^ culture dishes incubated at 37°C until reaching 70–80% confluence. At this point, the culture medium was refreshed and TmHg and EtHg (0.5; 1; 2 µM) were added and incubated at 37°C for 24 h, after which cells were collected by trypsinization and washed with PBS 1x. Subsequently, the pellet was resuspended in lysis buffer (25 mM TrisCl, pH 7.5; 100 mM NaCl; 2.5 mM EDTA; 2.5 mM EGTA; 20 mM NaF, 1 mM sodium orthovanadate, 20 mM sodium pyrophosphate; 20 mM of sodium *β*-glycerophosphate, 0.5% Triton X-100, and 1 tablet of protease inhibitor cocktail per 10 ml; Roche) vortexed and frozen at −20°C. These lysates were compared to a control group and used to quantify TrxR and Trx activity and expression as described below.

### Cell Fractionation

To obtain the mitochondrial and cytosolic fractions, after exposure to Hg compounds cells were washed and suspended in mitochondrial isolation buffer (210 mM mannitol, 70 mM sucrose, 1 mM EDTA, 10 mM Hepes–NaOH, and pH 7.5), containing protease inhibitor cocktail (Roche). Cells were disrupted with a Teflon pestle and then centrifuged at 600 g for 10 min at 4°C. The resulting nuclear pellets were discarded, and the supernatant was further centrifuged at 13,000 g and 4°C for 15 min, to obtain the mitochondrial pellets and the supernatant soluble fraction. Mitochondrial pellets were treated with cell lysis buffer as described above for whole-cell lysates and the supernatant fraction was further centrifuged at 100,000 g and 4°C for 1 h, to produce the cytosolic fraction (Branco et al., 2014). These fractions were used to quantify TrxR and Trx activity and expression as described below.

### Protein Quantification

The total protein present in the samples was quantified by the Bradford method. In a 96-well microplate (Nunc), samples were incubated with Coomassie dye (Bio-Rad; diluted 5 times) and subsequently, absorbance was measured at 595 nm in the microplate reader (Zenyth 3100, Anthos Labtec Instruments). Protein concentration in each sample was calculated from a calibration curve using BSA as a standard (Bradford, 1976).

### TrxR and Trx Activity Determination

The enzymatic activity of TrxR and Trx was determined according to the insulin end-point assay described by Arnér and Holmgren (2000) with modifications (Branco et al., 2014).

To determine TrxR activity, samples (50 µg for total cell lysates and 20 µg for subcellular fractions) were incubated in 96-well plates (Nunc) in TE buffer (50 mM Tris pH 7.5 + 2 mM EDTA) with 3 μM of human Trx (IMCO Corp, Sweden) (previously reduced with dithiothreitol, DTT, at 37°C and desalted in a NAP-5 column) and a master mix (1.6 mM insulin, 50 mM NADPH, 2 mM EDTA and 200 mM HEPES pH 7.6) for 20 min at 37°C. In parallel, control wells were prepared to contain the same reagents previously mentioned apart from Trx. After the incubation period, 250 µL of a 1 mM DTNB solution in 6 M guanidine-HCL (pH 8.0) was added to wells, and absorbance was measured at 412 nm in a microplate reader (Zenyth 3100, Anthos Labtec Instruments). The quantitation of TrxR activity was performed considering the difference in absorbance between the Trx-containing wells and the control wells. The same procedure was done for the determination of Trx activity, but the samples were incubated with 100 nM of recombinant rat TrxR (IMCO Corp, Sweden) instead of Trx (Branco et al., 2014).

### Expression of Proteins TrxR1, TrxR2, Trx1, and Trx2

The expression of thioredoxin system enzymes was evaluated by Western Blot in cell lysates and subcellular fractions after the separation of total soluble proteins (30 μg for both TrxR and for Trx) by SDS-PAGE on a 4–12% Bis-Tris gel with MES running buffer under reducing conditions (140 V for 1 h). Next, they were transferred to a nitrocellulose membrane (30 V for 2 h) and blocked with a 5% skimmed milk solution (1 h). The following antibodies were used: TrxR1 rabbit polyclonal IgG (sc-20147, Sta. Cruz), TrxR2 mouse monoclonal IgG (sc-376868, Sta. Cruz), Trx1 rabbit polyclonal IgG (ATRX8, IMCO Corp.), Trx2 rabbit polyclonal IgG (sc-50335, Sta. Cruz), GAPDH rabbit polyclonal IgG (sc-25778, Sta. Cruz), VDAC mouse monoclonal IgG (sc-390996, Sta. Cruz), goat anti-mouse IgG-HRP (sc-2005, Sta. Cruz), and mouse anti-rabbit IgG-HRP (sc-2357, Sta. Cruz). Expression levels were normalized for protein loading on the gel, which was assessed either by Ponceau-S staining prior to the blocking step or by evaluating the expression of housekeeping proteins (GAPDH, VDAC) (Branco et al., 2014).

### Evaluation of ROS Level

The assessment of general ROS production was based on the 2′,7′-dichlorodihydrofluorescein diacetate (H_2_DCFDA) assay (Huang et al., 2014).

Cells (8 × 10^4^) were plated in black 96-well microplates and then incubated at 37°C for 24 h. Afterwards, the medium was removed, the cells were washed twice with PBS 1x, and culture media with DHCF-DA (50 mM) was added to each well followed by a 40 min incubation at 37°C. After the incubation period, the DHCF-DA containing media was removed and the wells were washed twice with PBS 1x before being replenished with fresh medium. This was followed by exposure of cells to Hg compounds (EtHg and TmHg 0.5; 1, 2.5; and 5 µM) for 3 h using 1 mM H_2_O_2_ as a positive control. The fluorescence signal was read in a microplate reader (Zenyth 3100, Anthos Labtec Instruments) using 495 nm as the excitation wavelength and 529 nm as the emission wavelength.

### Trx Oxidation State (Redox Western)

The redox state of Trx1 and Trx2 was evaluated by the quantification of the number of reduced -SH groups (Free -SH), according to the Protein Electrophoretic Mobility Shift Assay (Bersani et al., 2002; Branco et al., 2017).

Briefly, after exposure to the TmHg and EtHg for 24 h, cell pellets were resuspended in Sample Solution (SS - Tris 50 mM, Urea 8 M, pH 8.3) containing 30 mM of iodoacetic acid (IAA; Sigma) and incubated at 37°C for 30 min to alkylate free thiols. This was followed by centrifugation at 13,000 g for 10 min, and the resulting supernatant was washed 3 times with an acetone/1 M HCl mix (98:2 v/v), followed by centrifugation at 13,000 g for 5 min. After evaporation of the acetone mixture, the resulting pellet was incubated at 37°C for 30 min in a SS solution containing 5 mM of DTT to reduce thiols oxidized by TmHg and EtHg. Posteriorly, these thiols were alkylated with SS containing 10 mM iodoacetamide (IAM; Sigma) at 37°C for 30 min.

Next, the samples were loaded onto a Urea-PAGE gel (stacking gel: 4% acrylamide; run gel: 12% acrylamide; 8 M Urea) and run at 10 mA. After 2 h, the proteins in the gel were transferred to a nitrocellulose membrane blocked with skimmed milk and incubated with respective antibodies - Trx1 rabbit polyclonal IgG (ATRX8, IMCO Corp.), Trx2 rabbit polyclonal IgG (sc-50335, Sta. Cruz) and mouse anti-rabbit IgG-HRP (sc-2357, Sta. Cruz). The migration pattern will vary according to the oxidation state of Trx, with more reduced enzymes being enriched in negatively charged IAA, thus migrating more in the gel than more oxidized enzymes, enriched in IAM. Mouse Trx1 presents a total of 6 Cys residues (2 in the active site plus 4 structural) and, therefore, this protein can assume 7 possible oxidation states varying between the totally reduced enzyme (6 free -SH groups) and total oxidation (0 free -SH groups). On the other hand, Trx2 presents only 2 Cys residues and 3 possible oxidation states (Branco et al., 2017).

### Prx2 Redox State

To evaluate the Prx2 redox state, cells were lysed in lysis buffer containing NEM (N-ethylmaleimide; Sigma) and, subsequently, the soluble protein fraction was separated by non-reducing SDS-PAGE electrophoresis, followed by transfer to a nitrocellulose membrane, after blockage with milk and incubation with the respective antibodies–Prx2 mouse monoclonal IgG (sc-515428, Sta. Cruz) and goat anti-mouse IgG-HRP (sc-2005, Sta. Cruz)—as previously described (Brown et al., 2008). The ratio between the bands of the Prx2 dimer and monomer was quantified for each sample using the Bio-Rad Quantity One software.

### Caspase-3 Activity

Caspase-3 activity was determined by measuring the enzymatic cleavage of chromophore p-nitroanilide (pNA) from the substrate N-acetyl-Asp-Glu-Val-Asp-pNA (DEVD-pNA) (Borralho et al., 2007).

This activity was determined based on a proteolytic reaction previously described (Borralho et al., 2007) with some modifications. In this assay total cell lysates were used, where cells were exposed to the TmHg and EtHg (1; 2 and 5 µM) for 24 h. This reaction was performed in an isolation buffer, containing 100 μg of cytosolic protein of cellular lysates, 50 μM of DTT, and 50 mM of DEVD-pNA (Sigma-Aldrich). Next, the reaction mixtures were incubated at 37°C for 3 h. Finally, the pNa formation was measured at 405 nm using a microplate reader (Zenyth 3100, Anthos Labtec Instruments) (Borralho et al., 2007).

### Guava ViaCount Assay

The ViaCount Assay was used to distinguish viable and non-viable cells based on the differential permeability of two dyes in the Guava ViaCount Reagent. The membrane-permeant dye stains all nucleated cells, leaving the cellular debris unstained, while the membrane-impermeant dye brightly stains damaged cells, thus indicating apoptotic and dying cells. GL261 cells (6 × 10^4^) were seeded for 24 h in 24-well plates and thereafter were exposed to the TmHg and EtHg for 24 and 48 h. Following treatment, cells were collected from plates along with cell culture supernatants and centrifuged for 5 min (650 g). Supernatants were discarded and the cells were resuspended in phosphate-buffered saline (PBS) containing 2% FBS. Subsequently, 15 μL of cell suspension were mixed with 135 μL of Guava ViaCount reagent and incubated for 5 min at room temperature. Sample acquisition and data analysis were performed with the Guava easyCyte 5HT flow cytometer (GuavaTechnologies, Inc., Hayward, CA, United States) using the ViaCount software module.

### Statistical Analysis

Results in tables and figures are presented as the mean ± standard error (S.E.) of at least 3 independent experiments. Statistical differences between groups were determined by applying a *t*-test for independent samples and considered significant at *p* < 0.05 and very significant at *p* < 0.01.

## Results

### Cytotoxicity Evaluation of EtHg, TmHg, and TMZ on Mouse GL261 Cells

**FIGURE 1:**
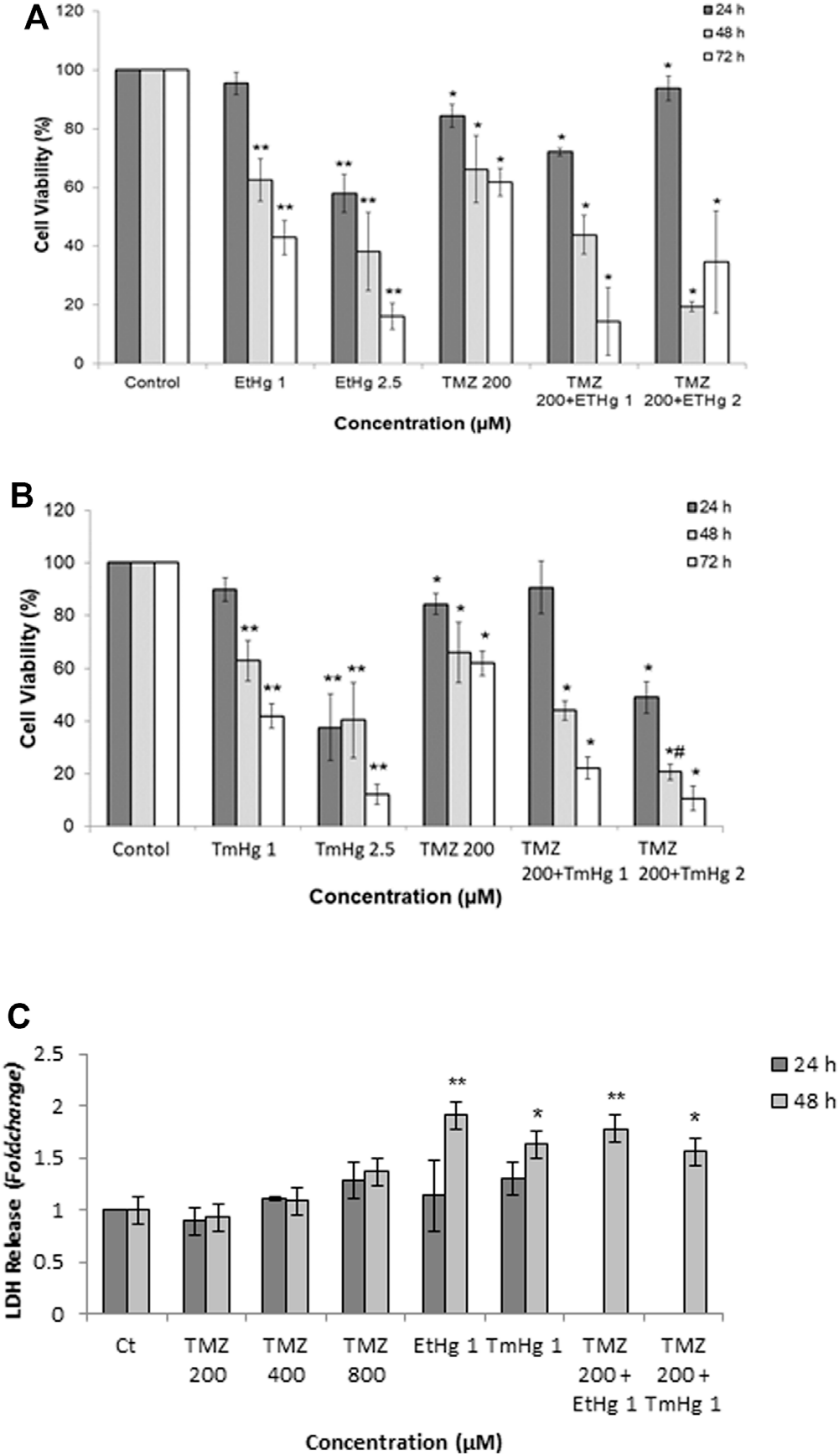
Comparison of cytotoxicity exerted on glioblastoma by mercury (TmHg and EtHg) and TMZ alone and in co-exposure with mercurials. Cell viability **(A, B)** and LDH release **(C)** of GL261 cells were tested upon exposure to different concentrations of compounds (EtHg and TmHg) for 24, 48, and 72 h. Cells were exposed to TMZ 200 µM and in co-exposure to 200 µM TMZ and 1 or 2 µM of EtHg/TmHg. Cell viability was performed by MTT assay **(A, B)** and LDH release into the culture medium was quantified **(C)**. Results are expressed relative to the non-treated control as the mean ± SEM of three to six different experiments. ^∗^ *p* < 0.05, ^∗∗^ *p* < 0.01 from non-treated control, #*p* < 0.05 from TMZ 200 µM.

The cytotoxicity of EtHg and TmHg to GL261 cells was evaluated by the MTT assay following 24, 48, and 72 h of exposure and confirmed by the LDH assay after 24 h of exposure (Figure 1).

Exposure to either TmHg or EtHg caused a concentration- and time-dependent inhibition of MTT reduction by GL261 cells which reflects a reduction in cellular viability (Figures 1A,B). As shown in Table 1 both mercury compounds caused an analogous effect on viability, with IC_50_ values at 24 h in the range (2.5–2.7) µM.

**TABLE 1:** Effects of TmHg, EtHg, and TMZ on GL261 and viability and death. All the determinations were performed three or more times in independent experiments.

Cell line		IC_50_*(µM)			EC_50_**(µM)	
		Exposure period			Exposure period	
GL261	Compound	24 h	48 h	72 h	24 h	48 h
	EtHg	2.7 ± 0.3	2.4 ± 0.4	0.97 ± 0.07	1.71 ± 0.4	—
	TmHg	2.5 ± 0.4	2.5 ± 0.4	0.92 ± 0.1	1.31 ± 0.2	—
	TMZ	—	444 ± 60	276 ± 7	—	>800

*IC_50_–calculated by MTT assay, **EC_50_–calculated by LDH assay.

TmHg and EtHg caused a similar LDH release and cytotoxicity (EC_50_ of 1.5 µM) on GL261 cells.

Additionally, the MTT assay was also carried out in GL261 cells exposed to different concentrations of TMZ. However, after 24 h exposure, TMZ failed to reduce viability by greater than 40% (*p* < 0.05) excluding IC_50_ calculation, which was only possible after 48 h and corresponded to (444 ± 60) µM (Table 1).

Based on these results the concentration of 200 µM of TMZ was used in the co-exposure assays with EtHg and TmHg.

Co-exposure assays showed (Figures 1A, B) that at all time-points, Hg compounds enhanced TMZ cytotoxicity in GL261 cells. This co-exposure effect was further confirmed in the LDH assay and the increase in LDH release is in good agreement with the results from the MTT assay.

On the other hand, as shown in Figure 1C, exposure to 200 µM of TMZ did not change the levels of LDH, but upon co-exposure with TmHg or EtHg (1 µM) LDH release increased by greater than 50% (*p* < 0.05).

### Effects of EtHg and TmHg on the Thioredoxin System

**FIGURE 2:**
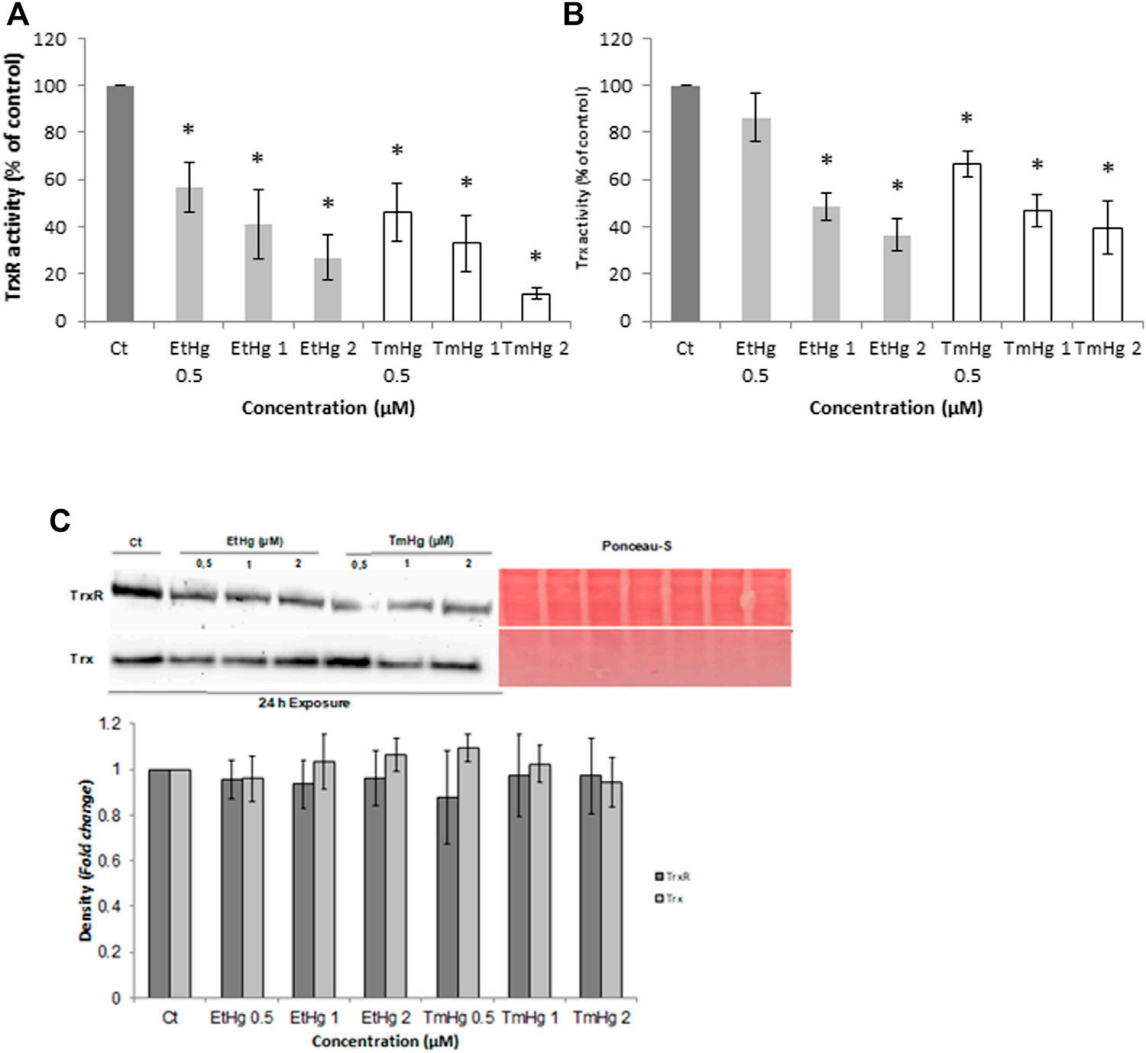
Effect of TmHg and EtHg on TrxR **(A)** and Trx Activity **(B)** and Expression **(C)**. Cells were exposed to mercury compounds (0; 0.5; 1 and 2 µM) for 24 h and enzymatic activity was measured by the insulin end-point assay in total cellular lysates. Expression levels were determined by Western Blot and results were normalized for protein loading with Ponceau-S **(C)**. Data in A and B are expressed as activity relative to the non-treated control. Values are the mean ± SEM of three to four independent experiments. ^∗^ *p* < 0.05 from non-treated control.

**FIGURE 3:**
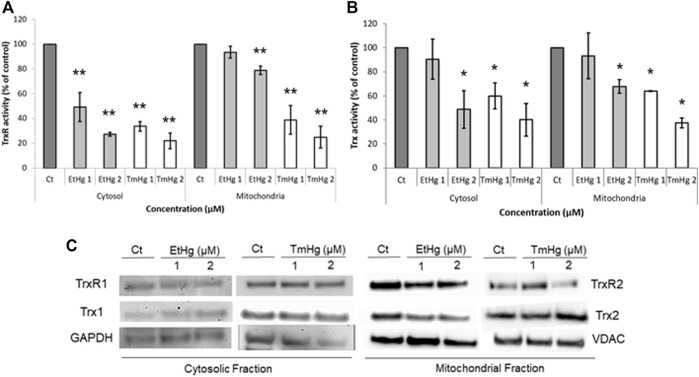
Effect of TmHg and EtHg on TrxR **(A)** and Trx **(B)** Activity and Expression **(C)** in the cytosol and mitochondria of GL261 cells. Cells were exposed to mercury compounds (1 and 2 µM) for 24 h and activities were measured by the insulin end-point assay in the cytosol and mitochondria after the separation of organelles as described in materials and methods. Expression levels were determined by western blots and results were normalized for protein loading on the gel with GAPDH and VDAC for cytosol and mitochondria, respectively, and Ponceau-S. Data in A and B are expressed as activity relative to non-treated control and the values are mean ± SEM of three to four independent experiments. Data in C demonstrate the blots. ^∗^ *p* < 0.05, ^∗∗^ *p* < 0.01 from non-treated control experiments.

EtHg and TM significantly inhibited the activity of TrxR and Trx in whole-cell lysates (*p* < 0.05) (Figures 2A,B) as well as in the subcellular fractions of cytosol and mitochondria (Figures 3A,B), in a concentration-dependent manner.

Considering the overall results, TrxR was more affected than Trx by both compounds. For instance, in whole-cell lysates, TrxR showed an IC_50_ of 0.8 and 0.7 µM (*p* < 0.05) for EtHg and TmHg, respectively (Figure 2), whereas Trx was inhibited with an IC_50_ of 1.3 µM (EtHg) and 0.7 µM (TmHg).

Thimerosal (1 µM) decreased TrxR activity by 60% (*p* < 0.01) in both subcellular fractions, whereas EtHg, while producing a strong inhibition over cytosolic TrxR (50% inhibition with 1 µM), had a lesser effect on TrxR activity in mitochondria (Figure 3A).

A comparable trend was observed for Trx activity, with thimerosal analogously affecting its activity in the cytosol (IC_50_ = 1.2 µM) and mitochondria (IC _50_ = 1.6 µM), and EtHg affecting the cytosolic activity to a greater extent.

Additionally, the expression of both enzymes in the two subcellular fractions was comparable (Figure 3C).

### Evaluation of Pro-Oxidant Effects Leading to Cytotoxicity

**FIGURE 4:**
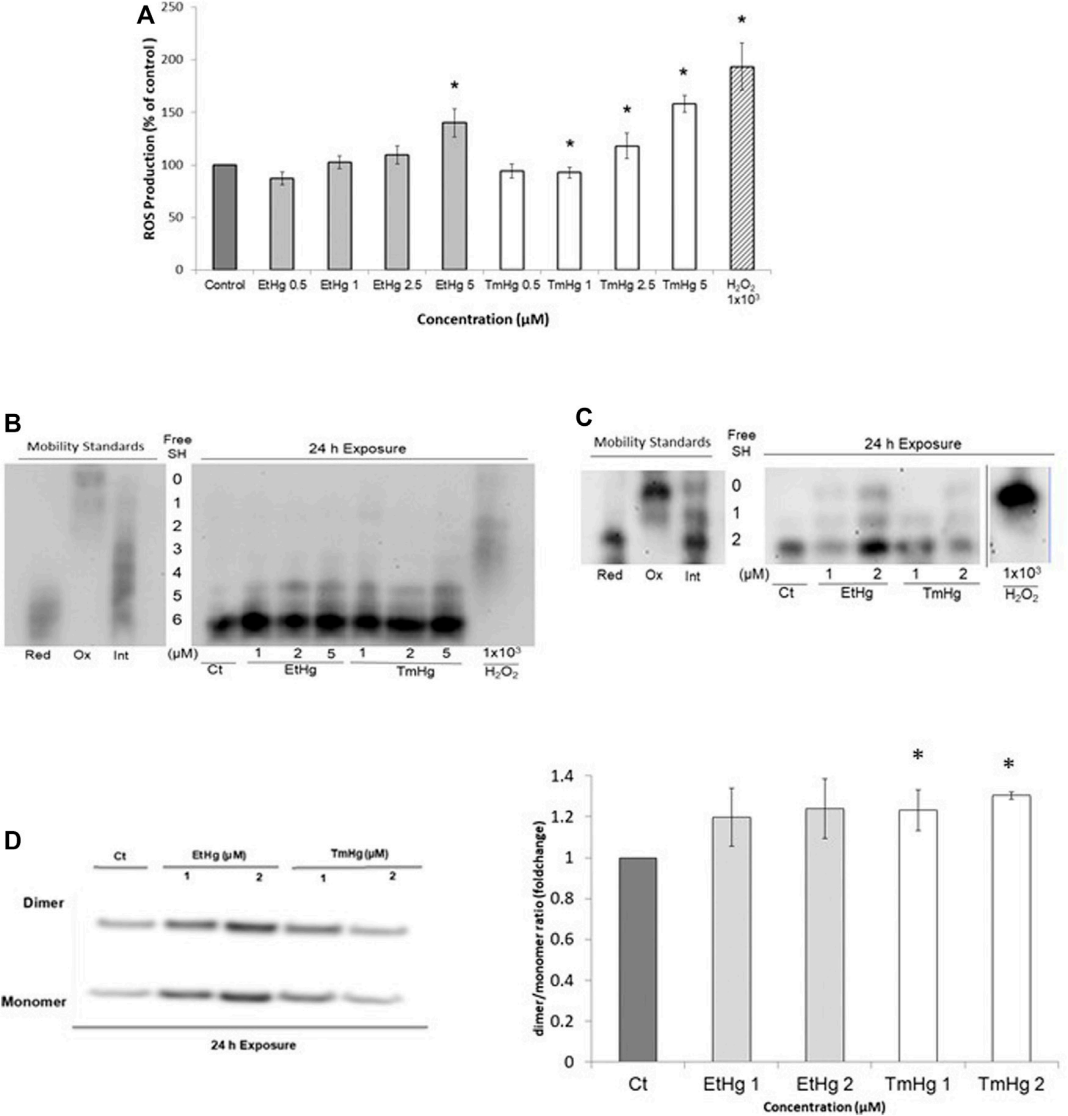
Pro-oxidant effects of exposure to EtHg and TmHg in GL261 cells. Cells were exposed for 3 h to 0; 0.5; 1; 2.5 and 5 µM of EtHg and TmHg and the ROS production was analyzed by DHCF-DA assay **(A)**. Concomitantly, the redox state of Trx1 **(B)** and Trx2 **(C),** and Prx2 oxidation **(D)** were evaluated by western blots after exposure to 0; 1; 2, and 5 µM (only Trx1) of EtHg and TmHg for 24 h exposure. Mobility standards correspond to the different oxidation states of Trx1 (7 possible states in relation to the presence of 6 Cys residues) or Trx2 (3 possible oxidation states related to the 2 Cys residues). Data are representative of at least 3 independent experiments. ^∗^ *p* < 0.05 from control. Red–fully reduced state; Ox–fully oxidized state; Int–intermediate oxidation states. The vertical line represents the site where the membrane was cut, corresponding to exposure to TmHg (5 µM) which due to the low sample amount could not be used to detect Trx2 by Western blot.

Upon EtHg and TmHg exposure there was a concentration-dependent increase in ROS production after 3 h of exposure, as compared with the control (Figure 4A). The pro-oxidant effects were slightly more significant with TmHg exposure.

As shown in Figures 4B,C, exposure to 1 µM of either EtHg or TmHg altered the redox states of Trx1 and Trx2 increasing the oxidation of Cys residues in both isoforms.

Also, exposure for 24 h of GL261 to 1 and 2 μM of both EtHg and TmHg caused a 20–40% increase in Prx2 dimerization relative to the non-treated control (Figure 4D), which was significant for TmHg exposure (*p* < 0.05).

### Caspase-3 Activity and Apoptosis evaluation

**FIGURE 5:**
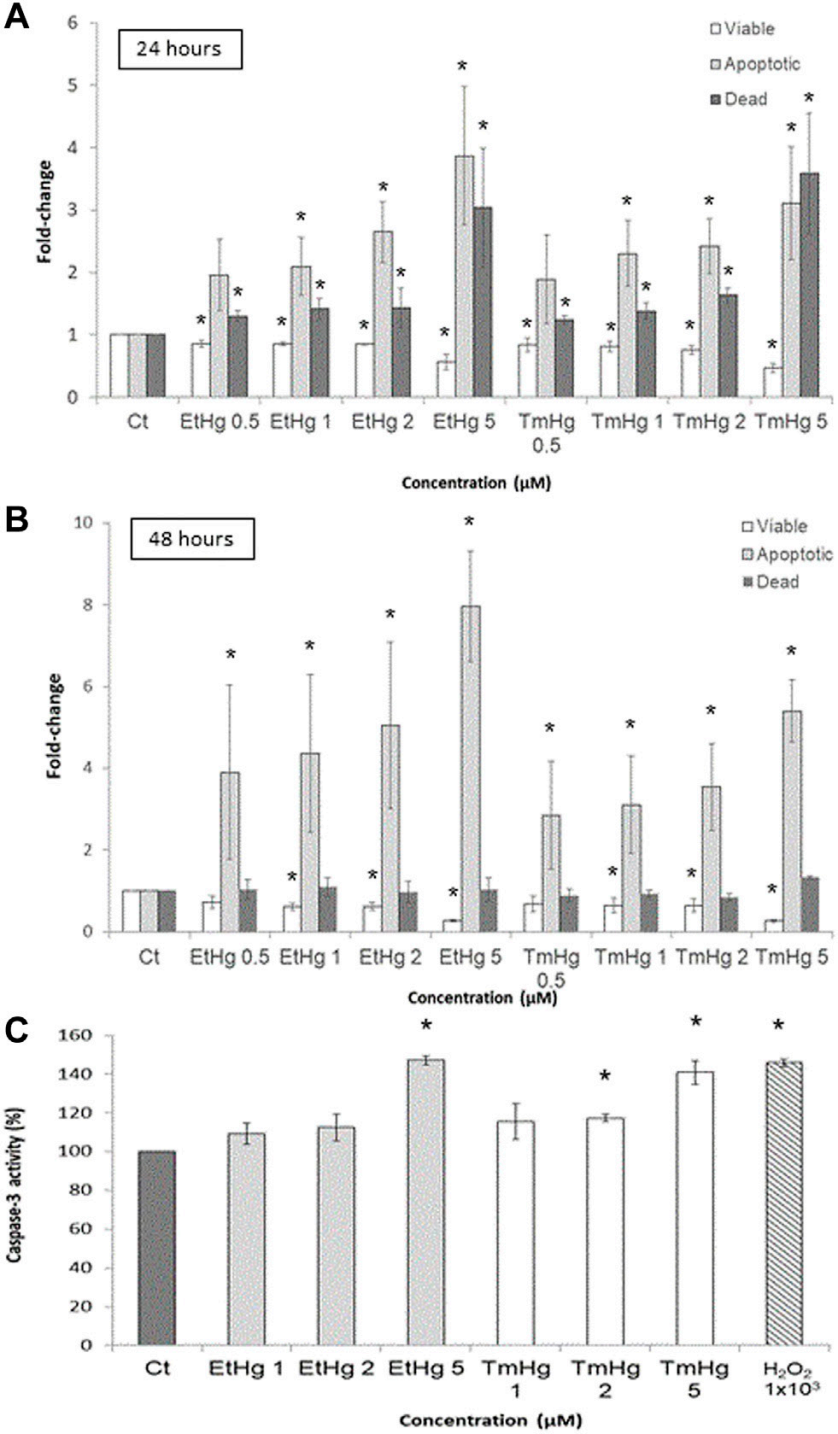
Cytotoxicity effects of EtHg and TmHg at GL261 mouse glioblastoma cells. Flow cytometry Guava ViaCount assay and Caspase-3 determination. The number of apoptotic cells following 24 **(A)** and 48 h **(B)** of treatment at different concentrations of TmHg and EtHg was determined by the Guava ViaCount assay. Results are expressed relative to the non-treated control as the mean ± SEM of three experiments ^∗^ *p* < 0.05 from the non-treated control. The activity of caspase-3 was also analyzed **(C)** by DEVD-pNA assay, where cells were exposed for 24 h to 1, 2, and 5 µM of EtHg and TmHg. Exposure to 1 mM H_2_O_2_ was used as a positive control.

Caspase-3 activity was increased by all exposure levels of both EtHg and TmHg up to 50% at 5 µM (*p* < 0.05) showing a concentration dependence (Figure 5C).

These findings are consistent with the Guava ViaCount assay which showed an increase (>50% when compared to the control group) in the number of apoptotic cells after 24 and 48 h of exposure to both TmHg and EtHg (Figures 5A,B). The increase in apoptotic cell numbers was concentration and time dependent.

## Discussion

This work aimed to evaluate the cytotoxicity of EtHg and TmHg in mouse glioblastoma cells as a consequence of disruption in the thioredoxin system. The results from the MTT assay revealed that GL261 viable cells decreased significantly with low concentrations of EtHg and TmHg, showing a similar IC_50_ corroborating the sensitivity of mouse glioblastoma GL261 cells to these mercury compounds.

**FIGURE 6:**
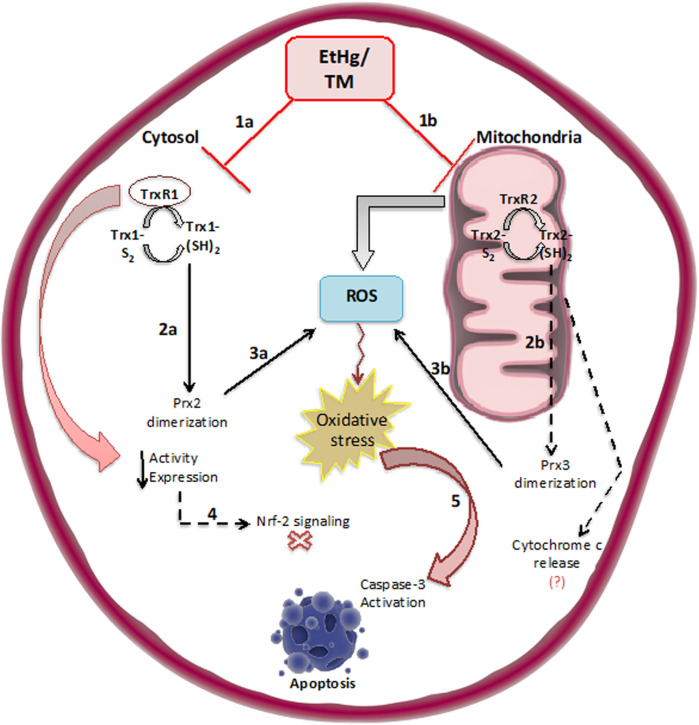
Schematic illustration of the effects of exposure to EtHg/TmHg on the Thioredoxin system both in the cytosol (TrxR1/Trx1) and in mitochondria (TrxR2/Trx2) (1a, 1b). As a result of Trx oxidation peroxiredoxins are dimerized (2a, 2b) causing a reduction in antioxidant capacity and accumulation of ROS (3a, 3b). At the same time, the reduction in TrxR/Trx activity and expression likely promotes Nrf-2 signaling as an attempt to recover enzymatic activity levels (4). Overall, oxidation of Trx, leads to caspase-3 activation, and eventually, apoptosis takes place (5). Nevertheless, some of these mechanisms need to be further confirmed, so the dashed arrows refer to those mechanisms such as analyzing the Nrf-2 signaling pathway, confirming Prx3 dimerization, and associated molecular events such as cytochrome c release, activation of caspase-8 and -9.

Notably, EtHg affected glioma tumor cells GL261, but to an extent lesser than on N9 microglia viability (data not shown), which is likely related to the higher vulnerability of cancer cells to oxidative stress (Figure 6). Similarly, TrxR and Trx inhibition by EtHg was significantly higher in GL261 than in microglia N9 cells (data not shown).

This effect on microglia is deemed profitable since the infiltration of microglia/macrophages in glioblastoma represents up to 30% of the tumor mass; thus, the regulation of microglial cell survival and proliferation will benefit tumor control (Lanza et al., 2021).

It was noteworthy that the co-exposure to 1 µM of EtHg/TmHg had a remarkable effect on cytotoxicity. These results imply that low concentrations of TmHg or EtHg alone or in co-exposure with TMZ significantly reduce glioblastoma’s cell survival (Figure 1).

The results on LDH release were congruent with the findings of the MTT assay, i.e. establishing heightened sensitivity of GL261 cells to Hg compounds and increased cell viability after the co-exposure of those compounds with TMZ 200 µM. Indeed, the EC_50_ for EtHg was 3-fold lower in GL261 cells than as previously reported in SH-SY5Y cells (Branco et al., 2017).

In agreement with previous findings from our group (Carvalho et al., 2008; Rodrigues et al., 2015; Branco et al., 2014), the activity of the Trx system was significantly affected by exposure to both EtHg and TmHg (Figure 2). TmHg, at levels below the IC_50_ decreases TrxR activity, thus stressing the importance of this system as a target for Hg (Carvalho et al., 2008).

Analysis of Trx system activity in subcellular fractions showed that TrxR1/Trx1 activities in the cytosol were significantly affected and to a greater extent in response to EtHg than TrxR2/Trx2 activities in the mitochondria. This is in agreement with earlier findings (Branco et al., 2017) in SH-SY5Y neuroblastoma cells exposed to EtHg and MeHg. On the other hand, TmHg similarly affected the Trx system enzymes at both cellular fractions. Thus, the TmHg showed greater toxicity than the EtHg in the mitochondrial fraction activity of both TrxR and Trx. This reflects TmHg greater tropism for mitochondria than EtHg, or alternatively, reflects an effect of the thiosalicylic acid resulting from its hydrolysis. In fact, it has been previously demonstrated that salicylates affect the mitochondrial membrane permeability, leading to the uncoupling of oxidative phosphorylation, which could justify the effect observed for TmHg (Miyahara and Karler, 1965).

Interestingly, TrxR2 expression was also reduced predominantly by the TmHg (Figure 3C). This is also of toxicological significance since TrxR2 is not regulated *via* Nrf-2, and therefore, the decrease in its levels may account for much of the activity decrease observed in the mitochondria (Branco et al., 2014).

Inhibition of the thioredoxin in the mitochondrial fraction promotes severe mitochondrial dysfunction and consequently leads to oxidative stress and impaired respiratory metabolism (Lopert et al., 2012; Sharapov and Novoselov, 2019). The increase in ROS levels is consistent with the results on enzymatic inhibition (Figure 4A) as well as the strong oxidation of Trx2 (Figure 4C).

In fact, exposure to EtHg and TmHg led to a change in the redox states of both Trx isoforms in GL261 cells (Figures 4B,C) after 24 h of exposure, consistent with previous observations in SH-SY5Y cells exposed to EtHg and MeHg (Branco et al., 2017). However, in the case of GL261 exposed to TmHg, this oxidation was most pronounced for Trx2, in agreement with the activity measurements.

Most importantly, Trx1 oxidation was reflected by increased dimerization of Prx2 (Figure 4D), consistent with the significant increase observed in ROS levels (Figure 4A), given that increased Prx2 oxidation will reduce the ROS-scavenging capacity of cells. In addition, given the strong oxidation of Trx2, it is expected that Prx3—which exists in the mitochondria and is a major substrate of Trx2—is oxidized, which has been previously observed in SH-SY5Y cells exposed to EtHg (Branco et al., 2017).

To further evaluate the effects of Hg compounds in GL261 cells, caspase-3 activity was quantified and found to significantly increase in response to treatments with 5 µM of organomercurials (Figure 5C). These findings along with the increase in the number of apoptotic cells observed in the Guava ViaCount assay suggest that cell death is occurring by apoptosis *via* the caspase-3 pathway. This is in agreement with results obtained by Branco et al. (2017), where SH-SY5Y cells exposed to EtHg showed increased caspase-3 activity associated with ASK-1 phosphorylation following Trx oxidation. In addition, Liu et al. (2007) observed in human gastric cancer cells (SCM1) that TmHg increased the activity of p38 and other members of the MAPKs family downstream of ASK-1 leading to increased activation of caspase-3. Thus, the current novel results indicate that the pro-oxidant effects of TmHg and EtHg causing Trx oxidation are linked to the triggering of GL261 cell death. It should be noted that Trx1/2 oxidation was already visible with exposure to 1–2 µM of either TmHg or EtHg, in agreement with the data on cytotoxicity.

## Conclusion

This study demonstrates the high sensitivity of mouse glioblastoma GL261 cells to TmHg and its metabolite, EtHg. Both TmHg and EtHg induced apoptotic cell death at low concentrations. While there is the need to further evaluate how other non-tumoral cells in the CNS cope with these compounds to prevent long-term neurotoxicity, as well as co-culture studies with microglia and astrocytes it should be stressed that presently glioblastoma has but a few therapeutic options with limited efficacy. Therefore, the potential of these compounds to be applied in GBM therapy should be further characterized and validated.

An important finding of this novel work was that exposure of GL261 cells to low levels of EtHg/TmHg resulted in significantly increased cytotoxicity when compared to TMZ alone. Given the lack of epidemiological evidence concerning TmHg neurotoxicity, consideration should be given to the repurposing of TmHg (Andresen et al., 2017) as a promising therapeutic approach for glioblastoma treatment.

## Data Availability

The original contributions presented in the study are included in the article/Supplementary Material; further inquiries can be directed to the corresponding author.

## Footnotes

1 WHO Technical Report Series 922 EVALUATION of CERTAIN FOOD ADDITIVES and CONTAMINANTS Sixty-First Report of the Joint FAO/WHO Expert Committee on Food Additives.
